# Aging gracefully: time and space matter

**DOI:** 10.18632/aging.204773

**Published:** 2023-05-25

**Authors:** Charline Roy, Laurent Molin, Florence Solari

**Affiliations:** 1 Université de Lyon, Université Claude Bernard Lyon 1, CNRS UMR 5284, INSERM U 1314, Institut NeuroMyoGène, MeLis, France

**Keywords:** DAF-2/insulin-IGF-1 receptor pathway, tissue-specific, muscle aging, lifespan

The main goal of research on aging is to understand the mechanisms responsible for the deterioration of organs and tissues with age, in order to counteract them and extend the health-span of human beings. In recent decades, the use of invertebrate model organisms has proven to be very useful in identifying genes whose mutations affect lifespan, thus helping to delineate molecular and cellular processes with a potential role in aging. Further studies in mammals have confirmed the involvement of the same mechanisms in aging and age-related diseases, leading to the identification of “universal hallmarks of aging” such as genomic instability, telomere attrition, epigenetic alterations, loss of proteostasis, deregulated nutrient sensing, mitochondrial dysfunction, cellular senescence, stem cell exhaustion, and altered intercellular communication. An important challenge is to understand how these hallmarks are coordinated between different tissues and with age. To address this question, we have recently developed a powerful tool in *Caenorhabditis elegans* to study the tissue- and age-dependent role of the DAF-2/insulin-IGF-1 receptor (IIRc) in aging [1]. The DAF-2/IIRc pathway is a paradigmatic example of a conserved pathway involved in aging from *C. elegans* to mammals [2]. Inactivating DAF-2/IIRc increases worm lifespan by 100 % and also prevents a plethora of age-related changes, including loss of mobility and resistance to stress. Previous results have indicated that the DAF-2/IIRc pathway functions in neurons and in the intestine to regulate lifespan [3–6]. However, whether age-associated *daf-2* mutant phenotypes rely on whole-body homeostasis and/or tissue- specific regulation had not been further investigated.

## DAF-2/IIRc pathway inactivation in gut or nervous system prevents life-limiting pathologies in *C. elegans*

We initially constructed a *daf-2* knock-in to visualize and deplete DAF-2/IIRc protein in a spatially- and temporally-controlled manner using an auxin-inducible system. Using this knock-in we were able to describe for the first time the expression pattern of DAF-2, which appears ubiquitous [1]. Inactivation of DAF-2/IIRc in neurons or gut only in adulthood was sufficient to extend the lifespan of the worms through shared downstream mechanisms, whereas depletion in epidermis, germline or muscle did not. In addition, a key feature of the health-span phenotype of *daf-2* mutants is their resistance to oxidative stress. Yet, our data suggest that while neuronal and intestinal DAF-2/IIRc affect lifespan through common mechanisms, those mechanisms do not seem to involve resistance to oxidative stress. Furthermore, neither intestinal nor neuronal depletion of DAF-2/IIRc prevented age-related loss of mobility. Thus, inactivation of the DAF-2/IIRc pathway in the gut or nervous system extends lifespan but does not necessarily delay the degeneration associated with aging of all tissues.

## Muscles integrate autonomous and non-autonomous DAF-2/IIRc-dependent signalling at different ages for the control of mobility

Mobility relies on the functional coordination of muscle and neuronal tissues. Down-regulation of DAF-2/IIRc only in muscle (which does not affect lifespan or resistance to oxidative stress) increases mobility in middle-aged worms to the same extent as *daf-2* mutation, while neuronal inactivation of DAF-2/IIRc does not improve the mobility of worms. In fact, neuronal inactivation of DAF-2/IIRc has the opposite effect as it decreases motility in early adulthood.

**Figure 1:**
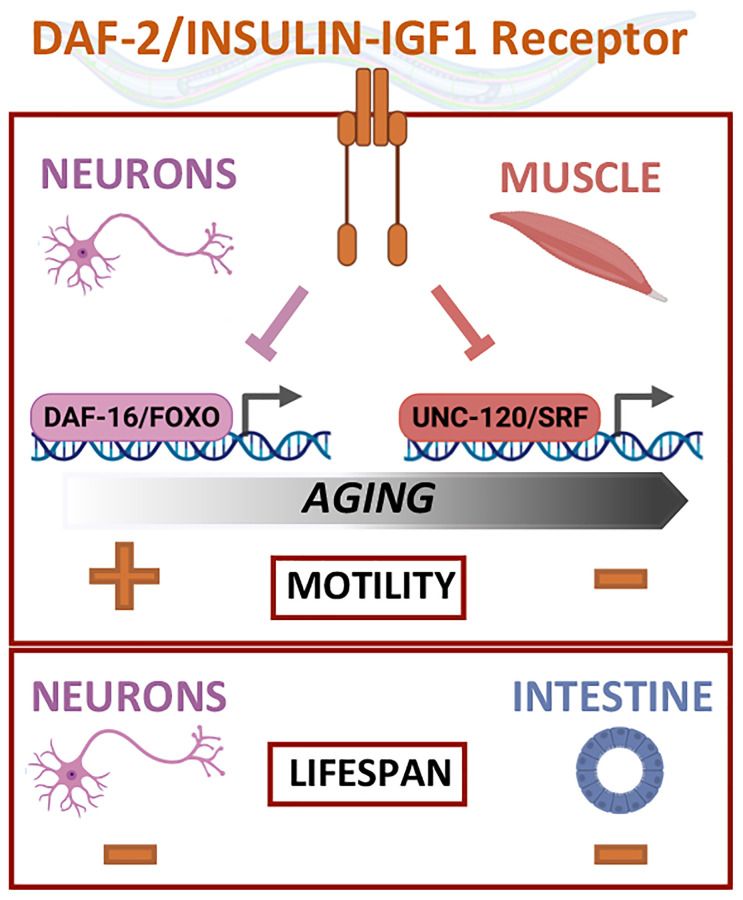
Regulation of lifespan and mobility by the DAF-2/insulin IGF-1 receptor relies on tissue-specific activities and effectors.

The canonical DAF-2/IIRc pathway involves the conserved FOXO transcription factor DAF-16 the activation of which is essential for the extension of lifespan when the pathway is down-regulated. However, the regulation of motility by DAF-2/IIRc does not depend on DAF-16/FOXO but requires the conserved MAD-box transcription factor UNC-120/SRF [1, 7]. In contrast, neuronal inactivation of DAF-2/IIRc decreases motility in early adulthood via DAF-16/FOXO (Figure 1), revealing the existence of tissue-specific effectors for the regulation of motility by DAF-2/IIRc.

We and others have shown that muscle aging is associated with progressive mitochondria fragmentation that is delayed in *daf-2* mutants [7]. Despite the antagonistic role of neuronal and muscular DAF-2/IIRc in the regulation of mobility, inactivation of DAF-2/IIRc in neurons or muscle prevents mitochondria fragmentation in muscle in old age. Thus, although a young mitochondrial network may be a prerequisite for the maintenance of the neuromuscular system with aging, the decrease in DAF-2 DAF-2/IIRc signalling in muscle regulates other factors necessary for the maintenance of motility that remain to be discovered.

Overall, results described in Roy et al. [1] show that the mobility pattern of *daf-2* mutants is determined by the sequential and opposing impact in the muscle tissue and neurons. This work provides the characterization of a versatile tool to analyse the tissue-specific contribution of insulin-like signalling in phenotypes associated to *daf-2* mutants. It also takes advantage of new tools for monitoring mitochondrial morphology and subcellular localisation of DAF-16/FOXO that avoid overexpression-related misinterpretation.

## Deciphering the complexity of aging through tissue-specific and time-controlled strategies

DAF-2 is the nematode ortholog of both IGF-1 and insulin receptors in mammals. Reduction in insulin or IGF-1 signalling pathways (IIS) also leads to an increase in lifespan, albeit more modest, while the mechanisms mediating the effects of IIS remain unclear. A recent study in mice reported that pharmacologic blockade of IGF-1R signalling preserves many aspects of health-span including neuromuscular function and attenuates the age-related systemic inflammation and neoplasia [8]. Paradoxically, IGF-1 has been shown to protect against osteoporosis, type 2 diabetes, cerebrovascular and cognitive decline in pre-clinical studies, as well as in some human epidemiologic studies. Coupling temporal and spatial analysis to pharmacological intervention should help determine the best protocol for pharmacological intervention.
